# “I must, and I can live with that”: a thematic analysis of patients’ perspectives on polypharmacy and a digital decision support system for GPs

**DOI:** 10.1186/s12875-021-01517-6

**Published:** 2021-08-21

**Authors:** Robin Brünn, Beate S. Müller, Benno Flaig, Petra Kellermann-Mühlhoff, Ute Karbach, Sara Söling, Christiane Muth, Marjan van den Akker, AdAM Study consortium, Lara Düvel, Till Beckmann, Reinhard Hammerschmidt, Julia Jachmich, Eva Leicher, Benjamin Brandt, Johanna Richard, Frank Meyer, Mathias Flume, Thomas Müller, Ferdinand M. Gerlach, Ana Isabel González-González, Truc Sophia Dinh, Kiran Chapidi, Peter Ihle, Ingo Meyer, Nina Timmesfeld, Hans Joachim Trampisch, Renate Klaaßen-Mielke, Jale Basten, Wolfgang Greiner, Bastian Surmann, Holger Pfaff, Juliane Köberlein-Neu, Alexandra Piotrowski, Karolina Beifuß, Sarah Meyer, Daniel Grandt, Simone Grandt

**Affiliations:** 1 Institute of General Practice, Goethe University, Theodor-Stern-Kai 7, 60590 Frankfurt am Main, Germany; 2 BARMER Statutory Health Insurance Company, Product Strategy and Product Development, Lichtscheider Str. 89, 42285 Wuppertal, Germany; 3 Department of Rehabilitation Sociology, Faculty of Rehabilitation Sciences, Technical University Dortmund, Emil-Figge-Str. 50, 44227 Dortmund, Germany; 4 Institute for Medical Sociology, Health Services Research and Rehabilitation Science, Department of Health Services Research, University of Cologne, Eupener Str. 129, 50933 Cologne, Germany; 5 Department of General Practice and Family Medicine, Medical Faculty OWL, University of Bielefeld, Universitätsstr. 25, 33615 Bielefeld, Germany

**Keywords:** Polypharmacy, Patient perspective, Qualitative study, Interviews, Decision support, Primary care

## Abstract

**Background:**

To investigate patients’ perspectives on polypharmacy and the use of a digital decision support system to assist general practitioners (GPs) in performing medication reviews.

**Methods:**

Qualitative interviews with patients or informal caregivers recruited from participants in a cluster-randomized controlled clinical trial (cRCT). The interviews were transcribed verbatim and analyzed using thematic analysis.

**Results:**

We conducted 13 interviews and identified the following seven themes: the patients successfully integrated medication use in their everyday lives, used medication plans, had both good and bad personal experiences with their drugs, regarded their healthcare providers as the main source of medication-related information, discussed medication changes with their GPs, had trusting relationships with them, and viewed the use of digital decision support tools for medication reviews positively. No unwanted adverse effects were reported.

**Conclusions:**

Despite drug-related problems, patients appeared to cope well with their medications. They also trusted their GPs, despite acknowledging polypharmacy to be a complex field for them. The use of a digital support system was appreciated and linked to the hope that reasons for selecting specific medication regimens would become more comprehensible. Further research with a more diverse sampling might add more patient perspectives.

**Trial registration:**

ClinicalTrials.gov, NCT03430336. Registered on February 6, 2018.

**Supplementary Information:**

The online version contains supplementary material available at 10.1186/s12875-021-01517-6.

## Background

Globally, the number of patients with polypharmacy, commonly defined as the routine use of at least five different drugs daily [1], is rising steeply, often because of multimorbidity [2]. The medical specialists treating the underlying diseases often use differing disease-specific guidelines, resulting in a complex and sometimes unbalanced treatment regimen [3, 4]. Furthermore, medical specialists often lack an overview of their patients’ medication, leading to duplicate prescriptions and treatments with antagonistic effects. Although polypharmacy is not necessarily harmful and many drugs may be required to treat multiple diseases, polypharmacy can become problematic when potentially inappropriate medications are used, or unrecognized side-effects treated with additional medicines [5]. Such mechanisms may result in overdiagnosis and overtreatment [6].

The number of possible drug-drug and drug-disease interactions rises exponentially with the number of drugs taken, possibly leading to therapy failure, worsening conditions, and reinforced effects. Computerized decision support systems (CDSS) that collect and process medical information and check for potential inappropriateness may help deal with this complex situation by indicating to GPs when an intervention might be appropriate. Such a tool and the effects of its use are evaluated in the AdAM study (“*Anwendung für ein digital gestütztes Arzneimitteltherapie- und Versorgungsmanagement*”, or “Application of an electronic medication support system”). In this study, a CDSS was implemented in participating general practices to help GPs assess and improve the therapy regimens of patients with polypharmacy.

From a patient perspective, the high treatment burden associated with polypharmacy may lead to non-adherence and inappropriate medication use [7, 8]. Simultaneously, the importance of including patients’ preferences in medication decisions is widely acknowledged [9]. Little is known about the role of patients in medication reviews, and the information that is available focuses on patients’ views on how they can participate in medication management [10] rather than on their perspectives on digital interventions to support polypharmacy care. Although facilitating pharmaceutical treatment, the digital support system evaluated in the AdAM study may raise a barrier between patients and their healthcare providers by reducing personal contact [11]. Further insight may help in the implementation of digital support systems to deal with polypharmacy, and, if effective, promote patient-centered care. Previous studies of patients that have received medication reviews have shown that patients tend to highlight certain changes [12]. Reviews are seen as beneficial when they involve clear communication and help increase the patient’s understanding of reasons for taking the drugs [13]. However, no digital medication review has yet been assessed.

This qualitative study aims to explore patients’ attitudes and beliefs with respect to coping with polypharmacy, their experiences of integrating polypharmacy into their daily lives, their views on GPs’ use of digital interventions to support medication management, and their concerns about potential negative consequences of the intervention.

## Methods

### Setting

This qualitative interview study was conducted as part of the AdAM cRCT that began in general practices in the German region of Westphalia-Lippe in 2017 and was completed in 2021. Its aim is to test whether a digitally supported annual medication review reduces hospitalization and death in polypharmacy patients. To be eligible for participation, patients had to be insured at the BARMER statutory health insurance company, 18 years or older, and have used five or more different medications for at least two quarters at the time of inscription. The complex intervention involved the use of a CDSS in which participating GPs could enter lab results such as kidney function, and claims data on medications and diagnoses. After the GP had entered the data, the CDSS assessed the medications and pointed out drug- and disease-related risks associated with, for example, dose, kidney function and possible interactions. (The CDSS is an expert-based system, i.e., a group of trained experts continuously screen medical publications, as well as notifications of German and international regulatory authorities that are based on the experiences of physicians and pharmacists. It encompasses all medication classes available in Germany. Evidence is systematically analyzed in accordance with the structured WHO UMC algorithm for the categorization of the causality of adverse drug reactions [14], and the quality of evidence appraised according to the GRADE system [15]). The GP then considered the assessment and generally discussed possible changes to the medication regimen with the patient and explained how the changes might be expected to affect him or her in terms of side effects and health improvements. Patients were thus involved in a shared decision-making process rather than in the application of the CDSS. The CDSS was available to physicians in practices randomly allocated to the intervention group. After a fifteen-month waiting period, control-group practices providing usual care switched to the intervention group. The full details of the trial’s design and methods will be published elsewhere.

### Population

We originally planned to select a purposive sample, whereby 200 patients participating in AdAM would be identified by BARMER, and stratified by age, sex, and postal code. These patients would be successively invited until a maximum of 20 interview participants had agreed to participate. The considerable organizational effort involved hampered patient recruitment. From January 2018 to October 2019, 15 GPs from the intervention group were asked to invite patients receiving the intervention to participate in this substudy. Interested patients received written information materials and a letter of consent to sign through their GP. To assist in recruitment, two reminders were sent to the practices.

### Material and methods

Information was collected using a semi-structured one-to-one telephone interview that was audiotaped and transcribed verbatim. We chose telephone interviews in preference to face-to-face interviews to minimize participation barriers. The interview guideline was broken down into four parts: medication, participation in the digital intervention, reflections on the intervention, and various comments. It consisted of 24 questions in total, not counting questions on socio-demographics (see web appendix). The interview guide was pretested on three representatives from the target population and adjusted accordingly.

From August 2018 to October 2019, either BSM or BF (both academic GPs, MD) called participants at home at a pre-arranged time in order to conduct the interviews. At the start of the interview and under guarantee of anonymity, participants were asked again if they would permit the interview to be audiotaped. Patients were aware of the interviewer’s identity, profession and role in the AdAM study. The researchers had no further contact with the patients.

### Data analysis

RB and MvdA used thematic analysis to analyze the interviews [16]. Regular discussion meetings took place when blocks of approximately three interviews had been analyzed. In our codebook, we decided upon codes in accordance with the themes used in our interview guide, which facilitated the initiation of data analysis. During the coding process, codes were altered and added when appropriate. Each researcher independently analyzed the interviews by coding sections according to the categories. Afterwards, coding was compared and discrepancies in findings resolved by discussion. After coding, all relevant citations were grouped according to subthemes in order to put them into context and highlight similar and differing statements. As well as being an effective way of coding, this deductive-inductive procedure allowed room for the addition of relevant codes, so that information outside the scope of the interview guide did not get lost. Illustrative quotes for each category were selected by RB and translated from German to English by a native English speaker. The translations were approved by RB and MvdA.

## Results

Thirteen interviews lasting between 11 and 56 (median 18) minutes were conducted. In one phone call, an interviewee was authorized to talk on behalf of her parents (marked with a footnote in Table 1), resulting in 14 cases in all, seven male (aged between 56 and 83, median 63 years) and seven female patients (aged between 62 and 88, median 81 years). The median number of drugs taken was six. Further information about the patients can be found in Table 1.

**Table 1:** Age, sex, residence, number of chronic prescriptions and other household members reported by participating patients

Patient	Age [years]	Sex	Chronic prescriptions	Other household members	Residence^b^
1	64	M	7	Two sons	Large city
2	88	F	6	None	Large city
3	82	F	5	None	Large city
4^a^	83	M	6	Spouse	Medium-sized city
5^a^	83	F	5	Spouse	Medium-sized city
6	66	F	9	Spouse	Medium-sized city
7	66	M	6	Spouse	Medium-sized city
8	58	M	7	Spouse	Medium-sized city
9	56	M	6	Spouse	Medium-sized city
10	60	M	12	None	Large city
11	81	F	8	Spouse	Medium-sized city
12	63	M	6	Spouse	Medium-sized city
13	62	F	8	Spouse	Medium-sized city
14	77	F	6	Spouse	Medium-sized city

^a^Interview conducted with their daughter

^b^Large city: population 100,000 + ; medium-sized city: population 20,000—< 100,000

Patients provided information on seven major themes:

1. Integration of polypharmacy in daily life
2. Use of a medication plan
3. Experiences with and attitudes towards own medication
4. Gathering medication-related information
5. Medication changes
6. Relationship with the GP
7. Patients’ perspectives of the intervention and the use of digital decision support tools

Data saturation was reached with P10, and the analysis of further interviews provided no additional information. We therefore decided against implementing a new recruitment strategy to achieve the initially planned sample size.

### Integration of polypharmacy in daily life

Overall, patients felt confident about dealing with polypharmacy. Although they often considered the number of their drugs to be high, it did not overwhelm them. Patients accepted that their medication belonged to their everyday lives, and organizing it became a ritual that they mostly performed weekly. Patients and family members generally prepared the medication without external help, while some engaged a nursing service.

*“I do things the way I learned from my wife—on Sundays, we prepare [the medication] for the week. […] Then I put them in those little boxes, and everything works flawlessly. […] Occasionally I forget, but that only happens once, maybe twice, a year.” (P7-min4).*

### Use of a medication plan

Federal health regulations permit patients with three or more medications to request a medication plan. Regardless of whether they had done so, medication plans were handed out to all study participants as part of the intervention, in most cases by their GP. Some kept them alongside their drugs and looked at them when preparing medications, while others ensured they were quickly accessible in case of emergency. Some patients always had their plan on them, e.g., on their phone or as a copy in each of their wallets, sometimes because they had previously been asked for it by staff in a hospital, pharmacy, or practice. Others said it reassured them to have it on them in case of situations in which health information might be crucial.

*“If I grab the wallet I need, I can be sure there’s a medication plan inside. […] When we went to Gran Canaria, the physician asked: ‘What medicines do you take?’ […] As there are so many, I haven’t got a clue when I’m agitated or whatever, […] but I can show him […]. This is what I take.” (P6-min6).*

Patients that had dealt with polypharmacy for some time barely used it if their medication rarely changed. Patients generally used it as a reminder when becoming accustomed to changes.

*“I don’t normally use [the medication plan] because […] I’ve been doing this for seven years now, so it’s stuck in my mind, and I know exactly when and how to take the tablets. I could tell you what they are too […]. But if you asked me ‘are they 5 or 10 mg…?’” (P9-min4).*

Patients found medication plans easy to read and helpful, especially after alterations, or when the brand name of a medicine had changed. Sometimes, patients struggled with updates and complained that they were not performed automatically, especially when other medical specialists were involved. This sometimes resulted in complicated situations when relying on the plan. One interviewee reported that the nursing service adhered strictly to the medication plan and refused to deviate from it without new written information.

*“If we didn’t ensure it was continuously updated by calling the practice and asking, […] the plans would be completely outdated. […] The fact that the specialists send their reports there is not enough to get them updated. […] Of course, they’re good because they give the nursing service certainty […] because [they] really watch out that […] the medication has been officially prescribed by the GP. They often refuse to provide anything that’s not explicitly written in the plan.” (Daughter of P4/P5 on their behalf-min11).*

### Experiences with and attitudes towards own medication

Patients described both positive and negative experiences with their medications. Interviewees accepted the need to take them, and not only spoke of having no problems with them but highlighted the positive effects of good adherence. Some were surprised that they didn’t experience side effects despite the quantity of their drugs and warnings about potential interactions by healthcare professionals.

*“I’ve never noticed anything, no nausea or skin redness or whatever else they describe. […] That’s what I sometimes find so astonishing. So far, I’ve tolerated all the drugs I’ve been given well. […] I notice myself that it really helps me. If I didn’t take my atenolol and amlodipine in the morning, my heart would start racing. […] As soon as I have the tablets inside me, everything’s ok.” (P8-min11).*

However, many patients did have unwanted reactions to drugs, some minor and seemingly easy to accept, and others severe or intolerable. Some patients felt overwhelmed by the contrasting views and contradictory advice of healthcare professionals. Pharmacotherapy becomes complicated when certain drugs are regarded as essential by one medical specialist but harmful by another, or when, despite a clear indication, drugs are not prescribed for fear of interactions. Both inappropriate polypharmacy and medication underuse occurred, with patients often having to choose one of two unsatisfactory options without clear advice from a trustworthy expert.

*“According to [the GP], taking […] Spasmex in the evening, as prescribed by the urologist for bladder cramps […], would clash with the other [drugs]. But the urologist says he cannot deprescribe it. It’s true that it causes mouth dryness, and that exacerbates problems swallowing. […] One says it’s necessary, and the other says it […] should be deprescribed. […] Problems swallowing is a major issue for [my father]. […] I’d like to get rid of it. But […] when he has cramps at night, it’s very unpleasant as well.” (Daughter of P4/P5 on their behalf-min5).*

*“There’s one drug that causes some constipation. But […] I must, and I can live with that.” (P10-min6)*

Patients found it confusing and displeasing when statutory health insurers exchanged a medication for a cheaper generic brand in order to comply with a federal health policy on cost-effectiveness. Apparently, the GP saw no particular reason to overrule the decision.

*“[For one drug,] additional costs of more than 200 euros per pack [first] have to be covered by the patient. And then one day, BARMER refused to reimburse the cost and doubted its necessity because there were [cheaper] alternatives, and [my mother] had always had bad experiences with them. […] And then, […] after she had bowel surgery […] and things were really life-threatening, she said: ‘I don’t care anymore, even if I’m reduced to poverty by this, I’m going to take […] the original brand again.’ And she says she tolerates it much better than other, generic drugs.” (Daughter of P4/P5 on their behalf-min20).*

Irrespective of good or bad experiences, some participants were skeptical about pharmaceuticals in general and saw them as a sign of bodily weakness to be avoided and only taken as a last resort. Others presumed the decision to begin pharmacotherapy was influenced by financial interests and questioned the use of some drugs.

*“I have to admit, I’d prefer to take none [of the drugs] at all. Because every drug you take is somehow an indication of weakness.”* (P7-min11).

*“If […] the [norm] levels are drastically lowered, half the country suddenly has a cholesterol problem because […] people want to make money out of it. I must admit that I ask myself whether it’s really necessary.” (P9-min12).*

### Gathering medication-related information

Most patients received information on their drugs from their physician or pharmacist and consulted them when questions arose. Some also asked relatives and friends, especially those working in healthcare. Few consulted the internet or hotlines provided by their health insurer.

*“I don’t have internet access. […] We have a very good pharmacy here. If I hadn’t asked the physician, I’d ask at the pharmacy […]. ‘What is this for?’ Because sometimes you hear that people at the pharmacy […] know more […] than physicians.” (P3-min26).*

*“My parents expect […] advice, but I often feel they’re asking too much of me. […] It’s hard for me to weigh up [interactions]. Fortunately, my sister is a pharmacist and so I always ask her.” (Daughter of P4/P5 on their behalf-min7).*

The directions for use were a controversial topic, with some patients reading them very thoroughly and checking for adverse effects and interactions, while others were frightened and confused by the warnings and preferred not to read them for fear of nocebo effects.

*“I don’t like [to read leaflets]. Because then there’d be loads of drugs I wouldn’t want to take. […] I think to myself: ‘Don’t worry about it.’ […] See how it works out.” (P6-min10)*

### Medication changes

Both patients and physicians initiated medication changes. When patients wished to stop taking a specific drug due to adverse effects, GPs generally followed their wishes.

*“I noticed that I feel much better when I don’t take [a specific drug]. I told [the GP] and since then, we have stopped it.” (P2-min6)*

When patients simply wanted to reduce the number of drugs, GPs were reluctant because they felt they were necessary. Patients accepted this decision once GPs had explained their views.

*“We tried twice. I asked [the GP] if father really needed the diuretic […] and each time […] after checking the lab results, she said: ‘No, he needs it for such and such a reason.’” (Daughter of P4/P5 on their behalf-min17).*

When lab results or the patient’s constitution encouraged physicians to deprescribe a medication, patients were appreciative.

*“I realized [my blood pressure] was very low. […] [My GP and I] talked about it and realized I was taking half a tablet too much. […] But we measured my blood pressure for several days first. […] And once, […] after taking a blood sample, the GP said […] my cholesterol level was normal again. […] Then we reduced [the medication].” (P7-min9).*

Patients rarely thought medication changes were attributable to the intervention.

*“The only thing that changed was [the treatment of high] cholesterol. The GP wasn’t content with [simvastatin] and therefore switched to atorvastatin. […] It’s supposed to act differently.” (P9-min15)*

Patients sometimes felt their conditions were worsening and asked for dosages to be raised.

*“I recently suggested taking more of the diuretic because my feet were swollen. That helped.” (P1-min4)*

### Relationship with the GP

All patients had great trust in their GPs and consequently in their prescribed therapies and the usefulness of the AdAM study. Patients wanted medical specialists to inform their GPs about their treatments because they viewed GPs as their key healthcare providers, whose advice had the highest priority. Acknowledging their own lack of knowledge, patients greatly appreciated their GP’s medication expertise. This trust manifested itself in the acceptance of a refusal to follow the patient’s desire to change a medication when the GP had explained why.

*“I have considerable trust in my GP. When he says: ‘You should take this’, […] I assume it to be true. […] I think it’s always better when people discussing things are experts rather than laypersons. And when my GP explains things afterwards, I understand it, too.” (P7-min19).*

*“He is my main contact in everything. That’s why I want him to be informed by other physicians.” (P9-min17)*

*“I asked [my GP]: ‘Can we deprescribe something?’ Because it is a lot. And he replied: ‘Actually, you need all the drugs.’ And that was that as far as I was concerned.” (P6-min8)*

### Patients’ perspectives of the intervention and the use of digital decision support tools

All interviewees were invited to participate in AdAM by their GPs. The main reasons for participating were the wish to support research, no expected negative consequences, and the desire to do their GPs a favor. Furthermore, patients hoped to contribute towards raising drug safety by avoiding interactions and reducing the number of their medications.

*“Afterwards I expect to be told what was discovered and [to hear] ‘Yes, we have this and that proposal for you’, perhaps regarding the intake of medications […] and what might be improved.” (P8-min13)*

*“I had the impression that [the GP] would appreciate it if she could convince me [to participate in the intervention] and that’s why I did it. […] [I had no expectations].” (P10-min9)*

Attitudes towards the digital intervention and its intentions were unanimously positive. Patients acknowledged that pharmacotherapy is a highly complex subject that physicians may not be adequately trained in, and they understood that by re-checking the medication, a computer program might prevent errors and provide additional information. One interviewee said the GP invested more time and effort in her case as a result, resulting in improved health. Most participants saw no changes, forgot about the study, or simply waited to see if something would happen. None of the interviewees reported any negative consequences such as fear, discomfort that healthcare providers were sharing information with others, inconvenience caused by the intervention, or concerns about the involvement of information technology.

*“Previously, we always had to deliver the information to the [general] practice, which was sometimes tedious. […] We now hope the information will reach the GP automatically, in its original form, and that she will have an overview. […] She said she took some extra time during the Christmas holidays to check all the medication. I thought that was extraordinary, and good. […] Previously, I rather had the impression I was responsible for pointing out difficulties. […] Now I think […] things are moving in the right direction by themselves. […] I can already see that the situation […] has improved.” (Daughter of P4/P5 on their behalf-min37).*

*“I thought it inspired confidence; because it’s a very complex subject. […] You can’t expect one person to know […] everything. The physician has her experience […], but it’s great that the computer does the preliminary work.” (Daughter of P4/P5 on their behalf-min29).*

Data protection was a concern, but the participants showed a high level of trust in all those involved and did not comment on the topic in detail. They were happy for their GPs to receive information from other healthcare providers, trusted the scientists using the data for research purposes and had confidence in the guarantee of anonymity. There was no resentment that a device interfered in the therapy, and no deterioration in the trusting relationship with their GPs. The fact that other physicians shared information with the GP was viewed positively.

*“That’s how things work in today’s digital world, and that’s why I said it was okay. It can be helpful for me as well. […] I don’t view it as my enemy.” (P6-min20)*

## Discussion

This study revealed that the interviewees had positive attitudes towards the digital intervention. Although encountering drug-related problems, patients seemed to cope well with their medication and had close relationships with their GPs. This lends support to previous studies investigating patients’ views on polypharmacy and patient-GP relationships. Clyne et al. found patients had disparate views on medicines, but the vast majority accepted medications as necessary and had trusting relationships with their GPs [17]. Moen et al. reported that adherence depended on the trust patients had in their GPs [18]. Uhl et al. also found that trust in GPs was the reason patients wanted them to conduct medication reviews. Concerns that GPs lack time and pharmacological competence were raised [10], but both could be mitigated through use of a CDSS.

Patients are aware that a lack of knowledge makes them dependent on the goodwill and expertise of their GPs. Although this implies their trust is the result of a knowledge imbalance [19], no participant considered this to be problematic. However, they noted difficulties obtaining plausible information and being understood, especially when polypharmacy-associated problems arose, or the opinions of healthcare providers conflicted with one another. The patients believed GPs provided the best treatment and prescribed the most suitable medications. Nevertheless, they experienced many drug-related problems. Patients expect digital tools to support GPs in the complex management of polypharmacy. They are aware that the subject is highly complex and that a CDSS has advantages over conventional care but cannot dictate medication changes to the GP, who maintains sovereignty. Since many intervention trials in healthcare fail to report harmful outcomes [20], we explicitly asked whether the GP’s use of a CDSS had any unwanted effects, but none of the interviewees reported this to be the case.

Family members may be overburdened when patients with polypharmacy rely on them for information. Since this finding could only be observed in the sole interview conducted with a caregiver, information was limited. However, the literature substantiates this view [21, 22].

This study has several limitations. Cases were picked from only few practices. However, a wide range of topics were extracted from the interviews. Furthermore, patients were recruited by their GPs, which may have resulted in selection bias if GPs only asked patients that were satisfied with the intervention to participate in the interviews. Patients that distrusted their GP would have preferred not to participate in the survey, and patients that declined to participate in the clinical trial from the beginning may also have had very different views from those that did, and perhaps have mistrusted the new digital decision support tool. Furthermore, as no patients from the waiting control group were interviewed, a comparison of the two groups could not be made here. Age and gender were balanced in our sample, but true purposive sampling could not be accomplished due to the small sample size. Therefore, our study can only give a restricted view on the topic but provides first insights that can be elaborated with larger and more diverse samples.

This is one of the first studies to investigate the views of patients with polypharmacy on digital decision support. Patients did not fear the use of a computerized system and appreciated the support it provided to the GP. Embedment in a clinical intervention is another strength since patients could report on their experiences directly. However, many patients had little knowledge about the intervention, and it is questionable to what extent they realized GPs were using a CDSS. On the other hand, this suggests that the digital tool was not perceived as disrupting the close patient-GP relationship, but might rather facilitate communication, as also indicated by a recent qualitative AdAM-substudy [23].

## Conclusion

As some patients find their therapy a burden, they hope and expect that the CDSS will facilitate their treatment and improve communication with and between medical specialists. Our study shows that this is also appreciated by patients, as they are generally in favor of physicians sharing information, and acknowledge pharmacists as a source of drug-related information. Other studies have shown that intensified interprofessional care involving physicians, pharmacists and nurses can improve the treatment of patients with polypharmacy [24–26].

Our analysis shows that the patients interviewed here manage their pharmacotherapy well despite drug-related problems. They see GPs as crucial figures in healthcare, in whom they have great confidence. Patients consider polypharmacy to be a complex field for physicians. They therefore appreciate computerized systems as a second source of information and a precautionary measure that supports GPs in their decision making. As digital tools are highly regarded by patients, their greater use in daily practice should be encouraged.

## Supplementary Information

**Additional file 1.**

## Data Availability

Data sharing is not applicable to this article as no datasets were generated or analysed during the current study. Transcripts of the patient interviews contain personal data and are therefore only available from the corresponding author on reasonable request.

## Abbreviations

AdAM: Application of an electronic medication support system
CDSS: Computerized decision-support system
cRCT: Cluster-randomized controlled trial
GP: General practitioner
P: Patient
