# Supplementary material for: “I must, and I can live with that”: a thematic analysis of patients’ perspectives on polypharmacy and a digital decision support system for GPs
Source: BMC Fam Pract. 2021 Aug 21;22:168. doi: 10.1186/s12875-021-01517-6 (PMC8379727; doi:10.1186/s12875-021-01517-6)
Supplement: Supplementary file 1 — Additional file 1. [file 12875_2021_1517_MOESM1_ESM.docx]

|  | **Topic** | **Questions** | **Notes** |
| --- | --- | --- | --- |
| 1 | **Welcome address** | **"Hi, my name is ________________.**  **I am __________________ (describe function at the Institute).**  **Thank you very much for agreeing to this phone call. As you know, today we would like to discuss things from the patient's perspective."**  If an appointment was agreed upon previously:  **"During our last phone conversation, we already spoke about our research project. Do you have any further questions?"**  If no previous appointment was arranged and the patient would like to be interviewed now, then continue to the presentation of the planned research project.  (Important: clearly differentiate between AdAM and the phone call) | □ Introduction  □ Introduction of AdAM |
| 2 | **Basic conditions** | **"The whole conversation will take around 20-30 minutes.**  **We are interested in your personal opinion on this subject, so there are no correct or incorrect answers. You are our expert on the subject of medication.**  **All your personal data will be anonymized so that it will be impossible to draw any conclusions about your person.**  **As mentioned before, I will tape record our conversation, so that we can better evaluate what you have said. Is that ok for you?"** [Turn on tape recorder.] | □ Consent to record conversation on tape  □ Turn on tape recorder |
| 3 | **Medication and medication plan** | 1. **"How many medications do you take?"** 2. **"How do they agree with you?"** (**"**Tell us about experiences you have had with your medications?**"**) 3. **"Tell us about a normal day (like today)? How do you organize taking your medications**? Do you prepare your day’s medications yourself, or does someone help you?” 4. **"What has been your experience of using a medication plan?"**   ("Some people make a mental note of the medications they are taking, while others write them down. How about you?  Where did you get the plan from? Did you write out the plan yourself or did you get it from your GP?”)   1. **"What does your plan look like?"**   Are black and white squares printed at the top of it?   1. **"What do you like or dislike about the plan?"** 2. **"What exactly do you do with the plan?"** (Where is the plan most of the time? Do you always have the plan on you? Who have you shown the plan to? Who has ever asked you about your medication plan?) 3. **"How do you tolerate your medications?"**   (Was that always the case? What did you do when it wasn't?  Is there anyone in particular that you talk to about such things?)   1. **"Tell us about any experiences you have had with the discontinuation of medications."** 2. Patient wanted discontinuation: **"What happens when you suggest discontinuing a medication to your GP?"** 3. GP wanted discontinuation: **"What happens when your GP suggests you discontinue a medication?"** 4. **"How do you find out about medications/side effects?"** (By calling a health fund hotline? On the internet?) 5. **"Are you taking a medication you don’t really want to take?"** 6. **"Are you taking any medications without the knowledge of your GP?"** | □ Introduction  □ Experience with medications  □ Medication management  □ Medication plan  □ Use of MP  □ Manageability  □ Practicability  □ Usability  □ Experiences of intolerance/ interactions  □ Deprescribing  □ Information needs  □ Compliance |
| 4 | **AdAM** | **Now I would like to talk about the AdAM project.**   1. **"Tell us how you found out about AdAM?"**   (How did you come to participate in it?)   1. **"When you decided to participate, what expectations did you have of it?"** ("How do you think it will affect you?" "How do you think it will affect your GP?") 2. **"What changes have come about for you since you have been participating in AdAM?"** 3. **"Tell us what happened when your GP last spoke to you about your medication?"**   (Were any changes made to your medication / were you given a new plan? In what situation was the new plan given to you? Did your GP talk about the new medication? More or less than before?)   1. **"How important is it to you that your GP knows about all your other treatments (e.g., when you have consulted other doctors?)"** (Were you ever confronted with a situation in which your GP knew something about you that you hadn’t told him/her? What did you think about that?) 2. **"What do you think about your GP using a special computer program like AdAM to check your medication?"** 3. **"Overall, does it inspire confidence, or is it rather uncanny?"** | □ Motivation  □ Expectations  □ Improvements  □ Disappointments  □ Communication GP-pat  □ Communication GP-specialist  □ Technology Acceptance Model (TAM)  □ TAM |
| 5 | **Reflection** | 1. **"What do you like about AdAM?”** 2. **"What don’t you like about AdAM? What disadvantages do you see for yourself and other patients that are participating in AdAM?"** (What reasons could you imagine for someone to decide not to participate in AdAM? Have you ever thought about withdrawing from the project? What treatments do you not especially like to talk to your doctor about? Or can you imagine that other patients might not like to talk about some consultations with their doctors? Would you like to prevent reports of your consultations with specific specialists from being automatically sent to your GP? 3. **"Would you recommend the AdAM project to your friends and family?"** | □ Supporting factors  □ Detrimental factors  □ Concerns/fears  □ Recommendation |
| 6 | **Conclusion** | We are slowly coming to the end of our conversation.   1. Is there anything else you would like to tell us? 2. Do you have any further questions, wishes or comments? | □ Final questions |
| 7 | **Sociodemo-graphics** | **"Finally, may I ask you to provide me with the following personal details…**  **□ Age**  **□ Sex**  **□ Nationality**  **□ Do you live alone or in a household with another or other person(s)?**  **□ Urban/rural (small town)/postal code**  **□ Family status: single, married, widowed, divorced**  **□ School leaving certificate**  **□ What is or was your profession?**  **□ Retired/working**  **□ Number of medications"** |  |
| 8 | **Thanks** | **"Thank you very much for participating in our research project and for contributing towards improving medical care."** |  |
